# Genetic variants of RNASE3 (ECP) and susceptibility to severe malaria in Senegalese population

**DOI:** 10.1186/s12936-018-2205-9

**Published:** 2018-02-05

**Authors:** Gora Diop, Céline Derbois, Cheikh Loucoubar, Babacar Mbengue, Bineta Niakhana Ndao, Fatou Thiam, Alassane Thiam, Rokhaya Ndiaye, Yakhya Dieye, Robert Olaso, Jean-Francois Deleuze, Alioune Dieye

**Affiliations:** 1 Faculté des Sciences et Techniques, Département de Biologie animale, Unité postulante de Biologie Génétique, Génomique et Bioinformatique (G2B), Université Cheikh Anta DIOP de Dakar, UCAD, Avenue Cheikh Anta DIOP, BP: 5005, Dakar, Sénégal; 2 Unité d’Immunogénétique, Institut Pasteur de Dakar, 36, avenue Pasteur, BP: 220, Dakar, Senegal; 3 Unité de Moyen-bas Débit, Institut de Génomique-CEA, Centre National de Recherche en Génomique Humaine, 2 rue Gaston Crémieux, CP 5721, 91057 Evry Cedex, France; 4 Faculté de Médecine, de Pharmacie et d’Odontologie, Service d’Immunologie, Université Cheikh Anta DIOP de Dakar, UCAD, Avenue Cheikh Anta DIOP, BP: 5005, Dakar, Senegal; 5 Département de Génie chimique et Biologie appliquée, Ecole Supérieure Polytechnique, Université Cheikh Anta DIOP de Dakar, UCAD, Avenue Cheikh Anta DIOP, BP: 5005, Dakar, Senegal; 6 Groupe G4, Biostatistique et Bioinformatique, Institut Pasteur de Dakar, 36, avenue Pasteur, BP: 220, Dakar, Senegal

**Keywords:** Severe malaria, *Plasmodium falciparum*, Susceptibility, RNase3 (ECP) gene, Polymorphisms, Senegal

## Abstract

**Background:**

Severe forms of malaria (SM) are an outcome of *Plasmodium falciparum* infection and can cause death especially in children under 4 years of age. RNASE3 (ECP) has been identified as an inhibitor of *Plasmodium* parasites growth in vitro, and genetic analysis in hospitalized Ghanaian subjects has revealed the RNASE3 +371G/C (rs2073342) polymorphism as a susceptibility factor for cerebral malaria. The +371 C allele results in an Arg/Thr mutation that abolishes the cytotoxic activity of the ECP protein. The present study aims to investigate RNASE3 gene polymorphisms and their putative link to severe malaria in a malaria cohort from Senegal.

**Methods/results:**

Patients enrolled from hospitals were classified as having either uncomplicated (UM) or severe malaria (SM). The analysis of the RNASE3 gene polymorphisms was performed in 241 subjects: 178 falciparum infected (96 SM, 82 UM) and 63 non-infected subjects as population control group (CTR). Six frequent SNPs (MAF > 3%) were identified, and one SNP was associated with malaria severity by performing a logistic regression analysis SM vs.UM: RNASE3 +499G/C (rs2233860) under age, sex as covariates and HbS/HbC polymorphisms adjustment (*p* = 0.003, OR 0.43, CI 95% 0.20–0.92). The polymorphisms: +371G/C (rs2073342), +499G/C (rs2233860) and +577A/T (rs8019343) defined a haplotype risk (G-G-T) for malaria severity (Fisher exact test, *p* = 0.03) (OR 4.1, IC 95% (1.1–14.9).

**Conclusion:**

In addition to the previously described association of +371G/C polymorphism in Ghanaians cohort, the RNASE3 +499G/C polymorphism was associated with susceptibility to SM in a Senegalese population. The haplotype +371G/+499G/+577T defined by RNASE3 polymorphisms was associated with severity. The genetic association identified independently in the Senegalese population provide additional evidence of a role of RNASE3 (ECP) in malaria severity.

## Background

Malaria is a disease that threatens more than one billion people worldwide and causes hundreds of thousands of deaths each year. A substantial reduction in morbidity and mortality occurred in endemic areas between 2000 and 2015 [1]. However, most cases still occur in sub-Saharan Africa. More than 80% of malaria clinical cases and around 90% of malaria deaths occur in sub-Saharan Africa, where the disease is responsible of 10% of the deaths in children under 5 years. Severe malaria (SM) corresponds to complicated forms of the disease, and globally 8–15% of affected subjects evolve to SM status [2, 3]. The mechanisms behind the fatal outcome of malaria are not fully elucidated. Severity factors have been suggested with both host and parasite genetic traits being proposed as major contributors. Evidence from other cohort studies revealed that 25% of changes from benign to severe forms of malaria are related to host genetic factors [4].

Most genetic studies have analysed immune factors implicated in inflammation, sequestration of parasite or vascular occlusion. For most factors studied, an association with SM was not clearly identified or conflicting results were reported [5]. Ribonuclease 3 (RNASE 3), also known as eosinophil cationic protein (ECP), is one of the factors suggested as having a role in malaria severity [6]. RNASE 3 is a single cationic polypeptide chain consisting of 133 amino acids with a high content of basic amino acids, particularly arginine. The gene coding for human RNASE3 is located on chromosome 14 (q24–q31) and the protein’s molecular weight ranges between 16 and 22 kDa, due to differential glycosylation levels with sialic acid, galactose and acetyl glucosamine residues [7, 8].

Patients with SM display hypereosinophilia and high levels of RNASE 3 [9]. Moreover, one study revealed that protein inhibited *Plasmodium falciparum* growth in vitro [10]. These observations led Adu et al. to perform a genetic analysis of the gene encoding ECP, which revealed an association of ECP polymorphism with cerebral malaria (CM) [11]. More specifically, the +371G/C polymorphism, resulting in an Arg/Thr substitution (to abolish ECP toxicity), has been associated with cerebral malaria in Ghanaian populations. However, this role of RNASE 3 +371G/C polymorphism on malaria severity was not reported in other studies.

In this work, RNASE3 gene polymorphisms was analysed in malaria patients from Senegal (West Africa). RNASE3 gene and its flanking regions were sequenced in samples from a cohort of urban individuals including healthy controls, uncomplicated malaria (UM) and SM subjects. Genetic variations, including single nucleotide polymorphisms (SNPs), were analysed among individuals, with respect to disease status in order to detect and confirm associations with malaria severity.

## Methods

### Malaria cohort and control subjects

The study population consisted of black Senegalese-born individuals whose parents and grandparents were born in Senegal and belonging to Wolof ethnic group. Malaria patients were enrolled from participating hospitals and corresponded to subjects with *Plasmodium*-positive Quantitative Buffy Coat (QBC), a test which is more sensitive than Giemsa-stained thick films [12]. The malaria patients were assigned to two groups: UM and SM, according to the criteria defined by Saissy et al. [13]. In brief, ‘severe malaria’ phenotype is characterize by the cerebral from, an organ failure and/or metabolic dysfunctions secondary to the presence of *P. falciparum* infection. Severe anaemia and cerebral form are the two most “severe form” currently explored, but other complications such as hypoglycaemia, thrombocytopaenia, renal insufficiency, hepatic or even pulmonary oedema may appear alone or in combination. Exclusion criteria included a travel, out of Senegal, in the 3 months prior to hospital admission; and display symptoms that might interfere with the study, such as recent pregnancy or childbirth or prior use of an anti**-**malarial treatment. The healthy control subjects corresponded to the exposed and uninfected subjects group in the same areas and belonging to Wolof ethnic group. A signed informed consent form was obtained from adult participants and parents or guardians of children involved in the study prior to blood sampling.

A total of 63 CTR, 82 UM and 96 SM subjects were included in the study (Table 1). This study was approved by the UCAD Ethic committee (University Cheikh Anta DIOP, Senegal) according the “Protocole 001l2015/CER-UCAD”.

**Table 1:** Demographic, clinical characteristics of patients and control group

Groups	Number of subjects	Age: mean (min–max) (year)	Sex groups			Hb*: mean ± SD (g/dl)	Parasite density* mean ± SD (P/μl)	Issue		
			M	F	ND			Survival	Died	ND
Control group (CTR)	63	38 (2–89)	29	23	11	*12.96* *±* *0.2*	*0*	63	0	0
Uncomplicated malaria (UM)	82	21 (0–74)	48	31	3	*12.11* *±* *0.2*	*3993* *±* *1327.3*	82	0	0
Severe malaria (SM)	96	25 (0–79)	60	28	8	*9.49* *±* *0.3*	*27220* *±* *5458.3*	63	25	8

*SM* severe malaria, *UM* Uncomplicated malaria, *CTR* Control group, *M* male; *F* female; *Hb* Haemoglobin, *ND* the number of patients, for witch, the sex and the issue were undetermined and/or unknowned. The number of patients showed in each group SM, UM and CTR. Age is given with median values (minimum and maximum values are in parenthesis). Hb levels and parasite density distribution are given with the mean and SD values (standard deviation). Hb* values statistically significant when compared SM to UM with p < 0.001. Parasite density* p < 0.001 when comparing SM to UM

### DNA extraction, RNASE3 gene, PCR and sequencing

DNA was extracted from whole blood using a Qiagen kit (QIAmp Kit Cat. n°51306) according to the manufacturer’s recommendations. The RNASE3 gene and its flanking sequences were amplified using the primers listed in Table 2. The PCR reactions were performed using a Gotaq^®^Green Master Mix (Promega, Germany) in a total volume of 25 µl containing 25 ng of genomic DNA (5 ng/µl) and 2.5 µl of each primer (10 μM). The PCR conditions were initial denaturation at 95 °C for 5 min, 35 cycles at 95 °C for 30 s, 62 °C for 45 s, and 72 °C for 1 min, with a final extension at 72 °C for 10 min. The amplicons were purified using BioGel P100 gels (Bio-Rad). Sequencing reactions (2 µl of PCR product) were performed using the dye terminator v3.1 method in an ABI PRISMs 3730 DNA Analyzer (Applied Biosystems, Foster City, CA, USA). Sequencing conditions were: 96 °C for 5 min, 25 cycles of 96 °C for 10 s; 60 °C for 4 min and 15 °C forever, and PCR products were purified with Sephadex G50 superfine columns (GE Healthcare). Alignment of acquired sequences and SNP discovery were performed using NC_000014.9 as a reference. Analysis was performed with Genalys version 2.0b software [14].

**Table 2:** Primers used to amplify the exons of RNASE3 by polymerase chain reaction (PCR)

Primers	Sequence (5′ > 3′)
RNASE3PromPF	GACAACCCCAGAACACACTG
RNASE3PromPR	AAGTGGGTCTCAGGTCTAGG
RNASE3Ex1PF	ACTATGCCTGCCTTCGTGTC
RNASE3Ex1PR	TTCCTTTACGCTGGGGTCTC
RNASE3Ex2PF	AACAATCCCCAGAGCTGGGA
RNASE3Ex2PR	GAGGGGGAGTTATAGACTGG

### Statistical analysis

Statistical analysis was performed to evaluate the association between malaria status and RNASE3 polymorphisms. For quality control of association analysis, we excluded the SNPs under the Hardy–Weinberg Equilibrium or with low frequency (MAF < 3%). Differences in allele frequencies among the three groups (SM, UM, CTR) were examined using logistic regression analysis. Linkage disequilibrium (LD) was computed for each pair of polymorphisms within the RNASE3 gene using Haploview software [15]. Haplotype estimates were obtained using the *Thesias* program [16]. Nominal p values were corrected under cofounders HbS and HbC polymorphisms, and respecting observed LD between markers. Associations with p values < 0.05 were considered statistically significant.

## Results

### RNASE3 gene variations and structure in Senegalese population

**Fig. 1:**
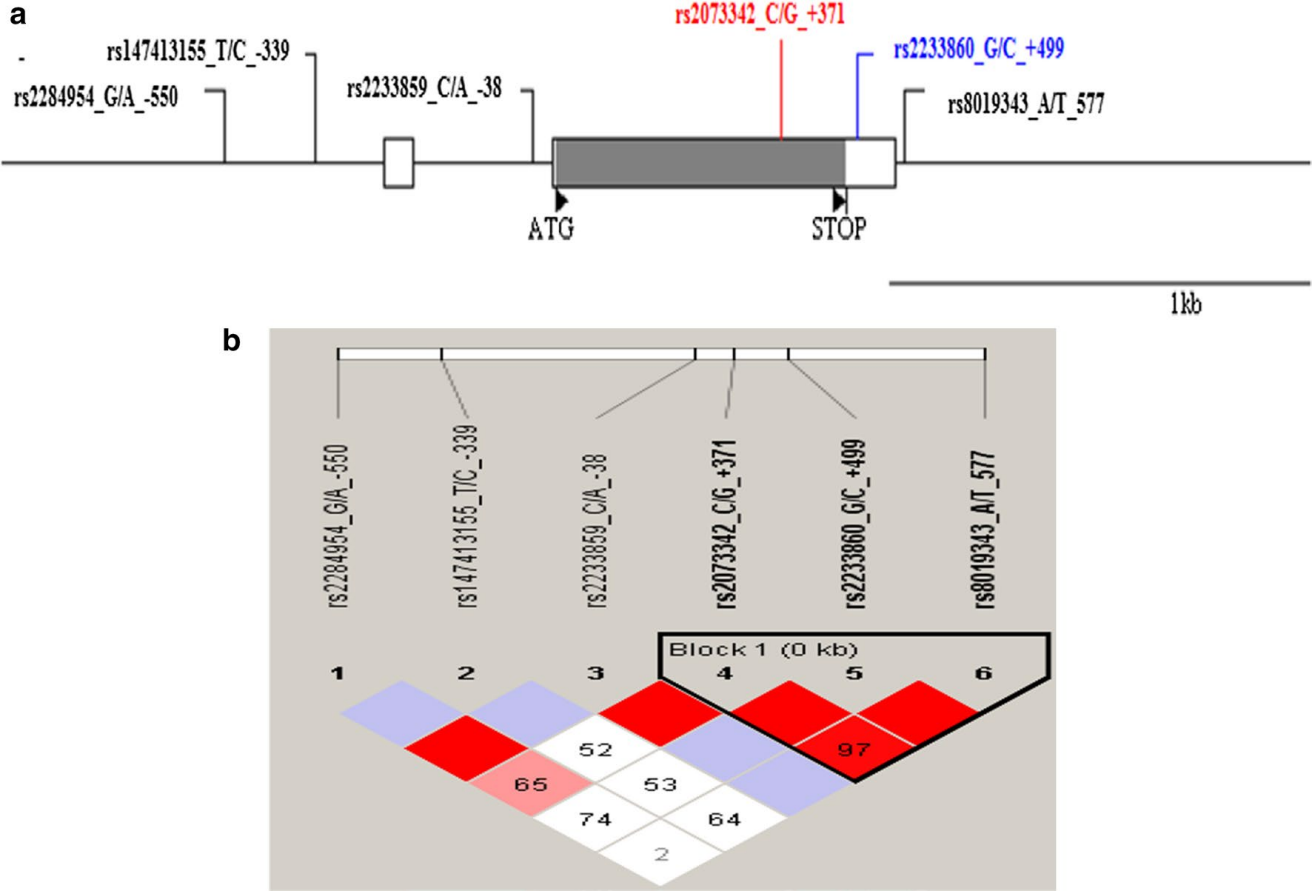
Structure and organization of *RNASE3* gene (ECP) located on chromosome 14q112 (14q24-q31). **a** RNASE3 gene polymorphisms located on chromosome 14q112 (14q24-q31). **b** Linkage disequilibrium map of the RNASE3 gene provided by the software Haploview for RNASE3 polymorphisms. **a** Coding and untranslated regions are indicated by black and white rectangles respectively. Positions are numbered according to the initiation codon ATG considered as + 1 (indicated by a black triangle). The genomic sequence used for alignment is GenBank sequence NC_000014.9-GI 568815584. **b** Linkage disequilibrium map of the RNASE3 gene provided by the software Haploview. The LD plot shows pairwise *D*′ values given in the squares for each statistical comparison between the SNPs. The different shade of color represent *D*′ values (between 0 and 1). An empty red square indicates that D′ > 0.97. The Lewontin’s D′ coefficient is correlated with the level of recombination: it is useful for the finding of an haploblock

In the present study, the polymorphism of the RNASE3 gene and its flanking sequences were analysed in a population of 241 Senegalese, including 96 SM, 82 UM and 63 CTR individuals. Six SNPs were identified including: 3 SNPs upstream the unique coding exon 2: RNASE3 –550G/A (rs2284954), RNASE3 –399T/C (rs147413155) and RNASE3 –38C/A (rs2233859); one SNP located within exon 2: RNASE3 +371G/C (rs2073342); and two located in the 3′UTR: RNASE3 +499G/C (rs2233860) and RNASE3 +577A/T (rs8019343) (Fig. 1a). Figure 1b shows pairwise linkage disequilibrium (LD) measured in each pair of polymorphisms. The values of D’ between SNPs were presented in Fig. 1b. For SNPs +371G/C and +499G/C we found D’ > 0.97 showing a high linkage disequilibrium. The presence of an haploblock structure in the RNASE3 gene was investigated and a genetic segment appeared to be part of a haploblock structure composed of 3 ‘successive’ RNASE3 SNPs, for which the confidence interval for D′ was 0.9–1 (+371G/C, +499G/C and +577A/T).

Table 3 summarizes the frequency of each polymorphism in the SM, UM and CTR groups. Allelic frequencies obtained in our study are similar to the data provided by the NCBI dbSNP database with regard to African populations except +577A/T. The SNP RNASE3 +371G/C introduces a non-synonymous change that results in an Arg/Thr mutation in protein sequence NP_002926.2, whereas +577A/T and +499G/C are located in 3′UTR.

**Table 3:** Allele frequencies and Hardy–Weinberg estimations of SNPs in the study population

Polymorphisms RNASE3					MAF (A1 frequency)				HWE* p value	Allele A1 African (NCBI db)	Allele A1 European (NCBI db)
Location referred to ATG meth start	NCBI dbSNP number	A1	A2	Amino acid change	SM	UM	CTR	Global population			
*Promoter* −550G/A	rs2284954	A	G	–	0.14	0.11	0.16	0.14	0.07	0.23	0.70
*Promoter* −399T/C*	rs147413155	C	T	–	0.08	0.03	0.03	0.04	0.02	0.04	0.00
*Promoter* −38C/A**	rs2233859	A	C	–	0.02	0.02	0.05	0.03	0.16	0.03	0.42
*Coding* +371_G/C	rs2073342	G	C	*Arg/Threo*	0.46	0.4	0.34	0.41	0.08	0.37	0.71
*3′utr* +499_G/C	rs2233860	C	G	–	0.14	0.25	0.23	0.21	0.15	0.23	0.18
*3′utr* +577_A/T	rs8019343	T	A	–	0.23	0.19	0.18	0.21	0.83	0.83	0.001

*SM* Severe malaria, *UM* Uncomplicated Malaria, *CTR* Control group *HWEp* Hardy–Weinberg in global group, *p* promoter region, *c* coding region, *u* untranslated region

* Deviation from Hardy–Weinberg, with values *p* < 0.012 (corrected threshold)

** Minor Allele frequency in Global population (*MAF*) < 0.05

### RNASE3 polymorphisms and severe malaria

Comparisons were performed among the three groups to test whether polymorphisms were associated with malaria severity. An analysis of Hb polymorphisms showed significant association with rs334 A/T and rs33930165 G/A mutations by comparison SM vs.UM and SM vs.CTR, respectively (*p* value = 0.033, OR 2.67 IC 95% 2.01–3.50) (*p* value = 0.0084, OR 1.67; 95% IC 0.99–3.42) (Table 4). Then, statistical RNASE3 polymorphisms analysis was performed using logistic regression tests with an adjustment for potential confounders such as Hb polymorphisms. The SNP +371G/C (Arg/Threo) yielded a borderline *p* value when comparing UM vs.CTR and SM vs.CTR: *p* = 0.08 (OR 1.8, 95% CI 0.35–0.91) and *p* = 0.05 (OR 1.78, 95% CI 0.99–3.1) in additive mode, respectively. The +499G/C polymorphism yielded a significant association with SM. For SM vs.UM, the +499G/C exhibit positives signals with the *p* values *p* = 0.003 (OR 1.43, 95% CI 1.20–1.92) and *p* = 0.001 (OR 1.28, 95% CI 1.10–1.76) in additive and dominant modes respectively. Considering both UM and CTR as unique control groups, the statistical comparison remain significant for +499G/C SNP with a p value *p* = 0.001(OR 1.50, 95% CI 1.30–1.84) and p = 0.004 (OR 1.29, 95% CI 1.21–1.74) in additive and dominant modes, respectively. The haplotypes were estimated using *Thesias* program. The triplotype estimation from 371G/C-499G/C-577A/T polymorphisms yielded a *G*-*G*-*T* positive signal with p values *p* = 0.03 when comparing SM vs. Controls (CTR +UM) (OR 4.1, 95% CI (1.1–14.9) (Table 5).

**Table 4:** Single Nucleotide Polymorphism (SNP) and association analysis with susceptibility to severe malaria (SM)

NCBI dbSNP number	Phénotype	MAF				Nominal p values for statistical test (additive models)		
		SM	UM	CTR	All	SM vs. UM	UM vs. CTR	SM vs. CTR
A. Hb S and Hb C polymorphisms and association with SM								
rs334 A > T *OR (95% CI)*	HbS	0.026	0.069	0.035	0.050	**0.033** *2.67 *(*2.01*–*3.5*)	0.257	0.446
rs33930165 G > A *OR (95% CI)*	HbC	0.000	0.009	0.029	0.011	0.258	0.1661	**0.0084** *1.67 *(*0.99*–*3.42*)
rs713040 C > T *OR (95% CI)*		0.107	0.068	0.119	0.099	0.097	**0.029** ***2.33*** (***1.55***–***7.33***)	0.660

RNASE3 SNPs		Nominal p values for statistical test							
Position (NCBI Ids)	Associated allele	UM vs. CTR		SM vs. CTR		SM vs. UM		SM vs. UM + CTR	
		ADD	D/R	ADD	D/R	ADD	D/R	ADD	D/R
B. Association analysis with susceptibility to severe malaria under Hb polymorphisms corrections									
*Promoter* −550G/A (rs2284954)	A	0.30		0.81		0.71		0.84	
*Promoter* −399T/C (rs147413155)	C	0.36		0.28		0.55		0.23	
*Promoter* −38C/A (rs2233859)	A	0.38		0.29		0. 98		0.47	
*Coding* +371_G/C (rs2073342) *OR (95% CI)*	G	0.08 *1.8* (*0.35*–*0.91*)	**0.01 (D)** *3.9* (*1.29*–*11.7*)	**0.05** *1.78* (*0.99*–*3.1*)	** 0.08 (D)** *2.18* (*0.88*–*5.36*)	0.86		0.17	
*3′utr* + 499_G/C (rs2233860) *OR (95% CI)*	C	0.52		**0.05** *1.46* (*1.21*–*2.09*)	** 0.01 (D) ** *1.30* (*1.11*–*1.79*)	**0.003** *1.43* (*1.20*–*1.92*)	**0.01 (D)** *1.28* (*1.10*–*1.76*)	**0.001** *1.50* (*1.30*–*1.84*)	** 0.004 (D)** *1.29* (*1.21*–*1.74*)
*3′utr* + 577_A/T (rs8019343)	T	0.66		0.16		0.17		0.06 *1.83* * (0.96–3.05)*	0.07(D) *1.98* *(0.92–4.32)*

For each polymorphism, the *p* values for statistical tests were indicated. Statistical tests used were logistic regression analysis. (A) Association analysis for HB polymorphisms by comparison of SM vs Controls group B) RNASE3 polymorphisms association analysis adjusted for HbS polymorphisms (rs334 A > T) (B). The associated allele of polymorphism is given. The calculation modes for logistic regression test are indicated using different models (*ADD* additive, *D* Dominant, *R* Recessive modes). Borderline (0.05 ≤ *p* ≤ 0.1) and significant (0 ≤ *p* ≤ 0.05) *p* values are in bold. The *p* values for the dominant/recessive mode were showed only when lower than 0.1. (* Bonferroni corrections were not performed on *p* values). The OR (odds ratio) and CI (Confidence intervals) were showed when *p* values are significant or in borderline and were reprented in italic

**Table 5:** Haplotypes frequencies estimations in the population and association with severe malaria (SM)

Haplotypes	Frequencies			Case vs. controls (Fisher exact test)
	Global	Case (SM)	Controls (UM + CTR)	
H1				
*CCA*	0.2	0.2	0.2	*p* = *0.19*
H2				
***GGT*** *OR (95% CI)*	0.17	0.24	0.14	***p*** **=** ***0.03*** *4.1 (2.1*–*14.9)*
H3				
*GGA*	0.08	0.12	0.07	*p* = *0.11*

Haplotypes analysis using *Thesias program*, and description of the association between cases SM (cases) vs. Control group (UM + CTR) using fisher exact test. Haplotype 2 was significantly associated with susceptibility to SM. *SM* Severe malaria, *UM* Uncomplicated malaria, *CTR* Control group. The significant *p* value of the haplotype H2 is in bold; *OR* 95% CI values were emphisized in italic format

### RNASE3 +499G/C (rs2233860) genotypes, severe malaria and biological parameters

Analyses were conducted to test whether RNASE3 +499G/C genotypes were associated with malaria severity. Comparisons of SM vs.UM showed significant association for 499 GC and +499 CC genotypes, yielding positives signals *p* = 0.006 and *p* = 0.013, respectively (Table 6). Taking into account of UM and CTR as unique control group, comparison of SM vs. UM +CTR exhibit an association for GC and CC genotypes with p values *p* = 0.002 and *p* = 0.04, respectively.

**Table 6:** Detailed association of RNASE3 +499G/C genotypes with malaria severity

Genotypes	Malaria Groups			Nominal p value for Statistical test							
	SM	UM	CTR	*UM* vs *CTR*		*SM* vs *CTR*		*SM* vs *UM*		*SM* vs *CTR* + *UM*	
				p	OR 95 %CI	p	OR 95 %CI	p	OR 95 %CI	p	OR 95 %CI
GG genotype as reference											
GG	70	41	31	–	1	–	1	–	1	–	1
GC	18	27	21	0.670	1.21 (0.49–2.08)	*0.014*	0.33 (0.14–1.02)	*0.006*	0.32 (0.14–1.82)	*0.002*	0.34 (0.17–0.98)
CC	4	7	2	0.210	3.15 (0.53–4.02)	0.365	0.37 (0.05–1.01)	*0.013*	0.11 (0.02–0.99)	*0.040*	0.18 (0.04–1.01)
CC genotype as reference											
CC	–	–	–	–	1	–	1	–	1	–	1
GC	–	–	–	0.305	0.38 (0.06–0.98)	0.921	0.89 (0.10–1.01)	0.246	2.86 (0.48–3.25)	0.480	1.82 (0.34–2.35)

This table gives RNASE3 u + 499G/C association analysis between groups. Significant p values are in italic. RNASE3 +499 GC and 499 CC genotypes induced significant association with severity. Logistic regression analysis were performed using plink analysis software. All the values OR were obtained with 95% CI

**Fig. 2:**
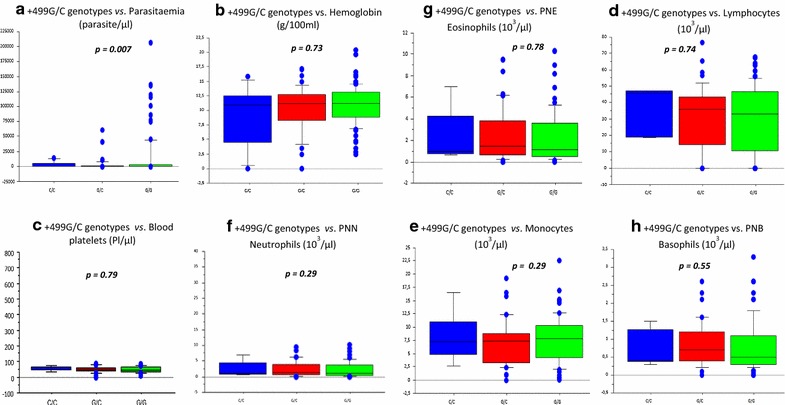
Associations between RNASE +499G/C genotypes and cohort biological parameters. Global comparisons of +499 Genotypes vs. biological parameters using Man Withney test. Significant association were showed with parasitaemia **a** with a p value *p* = 0.007. GG genotype (in blue color), GC genotype (in red color), CC genotype (in green color). *PNN* Neutrophil polynuclear; *PNB* Basophil polynuclear; *PNE* Eosinophil Polynuclear

Correlation of +499G/C genotypes and biological parameters (such as parasitaemia, survival/death and biological factors, including haemoglobin levels, blood platelets, lymphocytes, monocytes, basophils, neutrophils and eosinophils) using the Mann–Whitney test yielded significant association with parasitaemia levels with *p* = 0.007 (Fig. 2). The higher parasitaemia levels were observed in GG genotypes group. For eosinophils cells, producing ECP (RNASE3) protein, the level of cells is lower in CC genotype group but the differences are not significant (*p* = 0.78, Fig. 2).

## Discussion

In this study, an analysis of the RNASE3 (ECP) gene were performed, in order to identify polymorphisms associated with severe malaria in the Senegalese population. Six SNPs, with a MAF greater than 3%, were founded, all of which had been previously described and referred to dbSNP number (Table 3). NCBI frequencies of polymorphisms in Africans emphasizes with data obtained in our study except +577_A/T, but the differences are not significant. In opposite in Caucasian population NCBI frequencies are low, suggesting strong selection pressure in malaria endemic region.

In order to identify associations with malaria severity, statistical analyses using logistic regression were performed, taking account the age, and cofounders polymorphisms as covariate factors. There were a borderline positive signal with severe malaria for the +371 G/C (rs2073342), which induces an Arg/Thr shift. In the other hand, a positive signal was observed with the +499G/C polymorphism located in 3′UTR by comparing SM vs. UM or vs. CTR groups. Additionally, genotypic distribution of RNASE3 +499G/C was then detailed and the +499GC and +499CC genotypes showed significant associations with severity (Table 6). Finally the haplotype computation, taking into account +371 G/C, +499G/C and +577A/T SNPs, yielded *G*-*G*-*T* positive signal (*p* = 0.03). Then the interesting findings in this study relate to the two polymorphisms SNPs RNASE3 +371G/C and RNASE3 +499G/C founded to be in strong LD (D’ > 0.97, see Fig. 1), and the discussion will focused on them.

In a previous study from Adu et al. [11] in Ghanaians population, +371G/C polymorphism exhibit an association with cerebral malaria (CM), and RNASE3 +449G/C was not associated to CM. In the present study, the polymorphism +371G/C indicates a weak effect on severity and only the SNP +449G/C remained associate to SM. The frequency of associated +371G allele in Ghanaians population (0.32) is not significatively different of the frequency in Senegalese population (0.41). The differences of association between the analysis of Adu et al. [11] and this study could be explain, at first, by the fact that both studies enrolled patients from two different ethnic groups and a potential population substructure can influence the levels of association. Another explanation is the recruitment/inclusion criteria. In the present study, the “severe malaria” phenotype includes “cerebral malaria”, “respiratory distress” and “severe anemia” criteria. Therefore, these symptoms could probably dilute specific association of +371G/C polymorphism founded in Ghanaians study. In addition, the “age” is a strong factor in relation to infection and malaria severity outcome in endemic areas. Moreover, children under 5 years are more susceptible to develop severe symptoms in endemic areas, and the recruitments from Adu et al. [11] are performed specially in children group from 0.5 to 13 years. And finally, in this present study, statistical analysis were performed under age and sex corrections, in order to define genetic susceptibility specifically.

The biological significance of +371G/C polymorphism association, in the particular context of malaria pathogenesis were explained by the potential new glycosylation site of Arg/Thr shift, affecting the cytotoxicity of ECP, and the adhesion of eosinophils to intracellular molecules such as ICAM-1 and to post-capillary vein obstruction during cerebral stages [11, 17–20].

Furthermore, a genetic association of the +371G/C polymorphism was assessed in relation to other diseases, such as *Schistosomiasis,* hepatic fibrosis and asthma [21, 22], in which the functional effects are elucidated, and as being due to cytotoxic activity in epithelial cells of the nose and lungs of subjects with allergic disorders [17, 23].

The present study reveals, for the first time, an association of +499G/C transversion with severe malaria. The detailed genotypic distribution of +499G/C showed that +499C allele is a susceptibility factor to malaria severity. Surprisingly, the +499G/C polymorphism was not associated to cerebral malaria in Ghanaian population study [11], although the frequency is equal to 0.2 in the two populations. Probably, the association identified in our analysis could be due to the difference in phenotype screening, as mentioned previously. There are several potential transcription factors for which interactions with gene sequences could be prevented by +499G/C polymorphism, and the potential relationship between 3′UTR RNASE3 and expression mRNA Level has been largely described, emphasizing a low ECP in cells from subjects with 499GC and 499CC genotypes. Then the +499 G/C polymorphism in the 3′ end of the UTR of ECP gene may determine the ECP content in human eosinophil [24], and could plays a role on the control of Plasmodium parasitaemia as revealed by an in vitro analysis. Moreover, functional effect of the 3′UTR region have been shown in other gene, such as IL-12 [25].

Taking into account these results on SNP +499G/C, correlations were conducted to detect association of genotypes with parameters such as parasitaemia, and biological factors (including haemoglobin levels, platelets, lymphocytes, monocytes, basophils, neutrophils and eosinophils). Significant correlations were identified between +499CC genotype with a weak parasitaemia, suggesting a protective effect of 499C allele.

## Conclusion

Finally, SNPs in the RNASE 3 gene are commonly described [26], and most of them are identified in this study: i.e. the RNASE +371G > C and 3′UTR RNASE3 +499G > C polymorphisms. Others studies showed a genetic relationship between RNASE3 SNPs with the expression of diseases such as allergic asthma [23, 27], parasite infectious and/or inflammatory diseases [21, 28]. For malarial disease specifically, +371 G/C (Arg/Thr) polymorphism showed an association with cerebral malaria [11]. This study emphasizes, in a borderline effect, the association of this SNP with severity. Additionally, a strong association of +499G/C with disease severity was identified for the first time by this study. Moreover, these polymorphisms defined an association of the risk *G*-*G*-*T* haplotype for malaria severity.
